# Psychotraumatic Impact of Severe Postpartum Hemorrhage

**DOI:** 10.1192/j.eurpsy.2026.12001

**Published:** 2026-07-31

**Authors:** N. Smaoui, A. Jedidi, I. Gassara, R. Feki, H. Hkim, M. Maalej, N. Charfi, M. Maalej, S. Omri, L. Zouari

**Affiliations:** 1 Psychiatry C; 2 Gynecology-Obstetrics Department, Hedi Chaker Hospital, Sfax, Tunisia

## Abstract

**Introduction:**

Postpartum hemorrhage (PPH) remains one of the leading causes of maternal morbidity and mortality worldwide, particularly in low- and middle-income countries. Beyond its physical consequences, severe PPH can represent a highly traumatic event for mothers, potentially leading to long-term psychological sequelae. In recent years, growing attention has been given to the association between obstetric complications and post-traumatic stress disorder (PTSD), yet data from North African and Tunisian contexts remain scarce.

**Objectives:**

The objective of this study was to determine the prevalence of post-traumatic stress disorder (PTSD) among women who experienced severe postpartum hemorrhage (PPH).

**Methods:**

A cross-sectional, descriptive, and analytical study was conducted between January and December 2024 among 49 participants who presented with severe PPH. Their deliveries took place in the Gynecology-Obstetrics Department of Hedi Chaker University Hospital in Sfax between 2017 and 2023.

PTSD was assessed using the *Post-Traumatic Stress Disorder Checklist for DSM-5* (PCL-5), with a diagnostic threshold set at a score ≥ 31.

Statistical analysis was performed using SPSS-26 software.

**Results:**

The mean age of participants at the time of delivery complicated by PPH was 31.16 ± 5.86 years (min = 19, max = 47). More than 95% of participants belonged to low and middle socio-economic classes (n=47), and more than two-thirds came from rural areas (n=33).

Gestational age ranged from 35 to 42 weeks, with 81.63% of deliveries carried to term (n=40), and 45% of deliveries (n=22) performed by cesarean section.

PPH required invasive management in 23 participants (46.9%), including 4 (8.16%) who underwent hemostatic hysterectomy. Additionally, 18 participants (36.7%) were admitted to intensive care.

PTSD assessment revealed a mean PCL-5 score of 31.81 ± 18.54 (min = 4, max = 71). Overall, 22 participants (44.9%) met the DSM-5 diagnostic criteria for PTSD.

Analysis of PCL-5 dimensions showed mean scores of 7.98 ± 5.59 for re-experiencing (min = 0, max = 18), 3.78 ± 2.53 for avoidance (min = 0, max = 8), 9.84 ± 8.53 for negative alterations in cognition and mood (min = 0, max = 28), and 9.61 ± 4.83 for hyperarousal (min = 0, max = 22).

**Conclusions:**

Severe PPH is associated with an increased risk of PTSD. Systematic assessment of post-obstetric PTSD is essential to ensure appropriate and preventive management.

**Disclosure of Interest:**

None Declared

